# The Involvement of CXC Motif Chemokine Ligand 10 (CXCL10) and Its Related Chemokines in the Pathogenesis of Coronary Artery Disease and in the COVID-19 Vaccination: A Narrative Review

**DOI:** 10.3390/vaccines9111224

**Published:** 2021-10-21

**Authors:** Mojgan Noroozi Karimabad, Nicholas G. Kounis, Gholamhossein Hassanshahi, Farzaneh Hassanshahi, Virginia Mplani, Ioanna Koniari, Ming-Yow Hung, Ali Esmaeili Nadimi

**Affiliations:** 1 Molecular Medicine Research Center, Research Institute of Basic Medical Sciences, Rafsanjan University of Medical Sciences, Rafsanjan 7717933777, Iran; mojgan.noroozi@yahoo.com (M.N.K.); ghassanshahi@gmail.com (G.H.); 2 Division of Cardiology, Department of Internal Medicine, University of Patras Medical School, 26500 Patras, Greece; 3 Faculty of Veterinary Medicine, Islamic Azad University Shahr-e-Kord-Branch, Shahr-e-Kord 8813733395, Iran; Farzanehhassanshahi12@gmail.com; 4 Intensive Care Unit, Patras University Hospital, 26500 Patras, Greece; virginiamplani@yahoo.gr; 5 Department of Cardiology, University Hospital of South Manchester, NHS Foundation Trust, Manchester M23 9LT, UK; iokoniari@yahoo.gr; 6 Division of Cardiology, Department of Internal Medicine, Shuang Ho Hospital, Taipei Medical University, New Taipei City 23561, Taiwan; myhung6@ms77.hinet.net; 7 Division of Cardiology, Department of Internal Medicine, School of Medicine, College of Medicine, Taipei Medical University, Taipei 110, Taiwan; 8 Taipei Heart Institute, Taipei Medical University, Taipei 110, Taiwan; 9 Department of Cardiology, Medical School, Rafsanjan University of Medical Sciences, Rafsanjan 7717933777, Iran; dr_esmaeili_n@yahoo.com; 10 Occupational Environment Research Center, Rafsanjan University of Medical Sciences, Rafsanjan 7717933777, Iran

**Keywords:** acute coronary syndromes, CXCL10, Chemokines, coronary artery disease, COVID-19, vaccines

## Abstract

Coronary artery disease (CAD) and coronary heart disease (CHD) constitute two of the leading causes of death in Europe, USA and the rest of the world. According to the latest reports of the Iranian National Health Ministry, CAD is the main cause of death in Iranian patients with an age over 35 years despite a significant reduction in mortality due to early interventional treatments in the context of an acute coronary syndrome (ACS). Inflammation plays a fundamental role in coronary atherogenesis, atherosclerotic plaque formation, acute coronary thrombosis and CAD establishment. Chemokines are well-recognized mediators of inflammation involved in several bio-functions such as leucocyte migration in response to inflammatory signals and oxidative vascular injury. Different chemokines serve as chemo-attractants for a wide variety of cell types including immune cells. CXC motif chemokine ligand 10 (CXCL10), also known as interferon gamma-induced protein 10 (IP-10/CXLC10), is a chemokine with inflammatory features whereas CXC chemokine receptor 3 (CXCR3) serves as a shared receptor for CXCL9, 10 and 11. These chemokines mediate immune responses through the activation and recruitment of leukocytes, eosinophils, monocytes and natural killer (NK) cells. CXCL10, interleukin (IL-15) and interferon (IFN-g) are increased after a COVID-19 vaccination with a BNT162b2 mRNA (Pfizer/BioNTech) vaccine and are enriched by tumor necrosis factor alpha (TNF-α) and IL-6 after the second vaccination. The aim of the present study is the presentation of the elucidation of the crucial role of CXCL10 in the patho-physiology and pathogenesis of CAD and in identifying markers associated with the vaccination resulting in antibody development.

## 1. Introduction

Cardiovascular disease (CVD) is defined as one of the leading causes of mortality, accounting for over 15 million deaths worldwide [1,2]. CAD is one of the most common forms of severe CVD and has a major impact on personal health accompanied with high mortality, hospitalization rates and healthcare expenses (approximately 312 billion USD per annum in the USA) [3,4]. According to the most recent studies, atherosclerosis—defined as a chronic inflammatory state affecting both the medium and large arteries—constitutes the main underlying cause of CVD [5,6]. The activation of the vascular endothelium, along with the accumulation of lipids, immune cells and cell debris, leads to atherosclerotic lesion formations prone to ruptures and thromboses as well as further partial or total arterial occlusions [5,7]. The progression and rupture of atherosclerotic lesions are predisposed to cardiac and cerebral ischemic events causing myocardial infarctions and strokes, respectively. White blood cells (WBC) play a crucial role in the initiation and progression of atherosclerosis [8,9,10] as a developing inflammatory disorder. Chemokines, a subgroup of the cytokine network, may serve as pro-migratory factors for a broad variety of cell types expressing the appropriate trans-membrane G-protein and specialized chemokine receptors. These low molecular weight cytokines are sub-classified into C, CC, CX3C and CXC based on the position of the conserved cysteine motifs within their biochemical structures [11,12]. In addition, based on the presence or absence of the enzyme-linked receptor (ELR) group (a motif containing glutamine-leucine-arginine adjacent to the cysteine residues), CXC chemokines are further divided in two subgroups: ELR, which stands for the three amino acid residues Glu-Leu-Arg (ELR), and the ELR− and ELR+ chemokines such as the CXC motif chemokine ligand 1 (CXCL1), CXCL2 and CXCL3, which act as neutrophil chemo attractants. ELR-CXC chemokines including CXCL10 and CXCL12 serve as pro-migratory agents of peripheral blood mononuclear cells (PBMC) such as lymphocytes, monocytes and natural killer (NK) cell types [13,14]. CXCL10 chemokine, known as interferon γ (IFN-γ)-inducible protein 10 (or IP-10) was initially discovered as an IFN-γ-induced protein produced by a wide spectrum of cell types varying from endothelial cells [15], monocytes [16,17], keratinocytes [18], neutrophils [19], dendritic cells [20] and fibroblasts [21] to hepatocytes [22], and mesenchymal cells [23,24]. The common but not specialized receptor for CXCL10 is CXCR3 [25]. The CXCL10/CXCR3 axis is essential for white blood cell (WBC) migration toward damaged and inflamed tissues, leading to further tissue injury [26]. Moreover, chemokines control the migration of immune cells toward infection/inflammation areas and injured tissues [24,27]. Beyond inflammation perpetuation and immune cell migration, chemokines are also involved in other functional activities such as new blood vessel formation, known as angiogenesis, or the inhibition of the former, called angiostasis [14,28]. More than 50 chemokines and 20 chemokine receptors have been recognized to date [29]. Multiple members of CXC chemokines have been confirmed to be involved in inflammatory conditions such as type-1 diabetes [30], Behcet’s syndrome, diabetes mellitus [31], osteoporosis [32], preterm delivery [33], cancer [34], systemic lupus erythematosus (SLE) and tick-borne encephalitis. However, a clear relationship between chemokines and CAD needs to be fully elucidated. The main goal of the present review is the elucidation and clarification of the role of CXCL10 as an inflammatory mediator for atherosclerosis progression and further CAD.

## 2. Biostructure and Functions of CXCL10

It has been well-documented that several chemokines, especially CXCL10, are generated and secreted by various cells and tissues, exhibiting pleiotropic effects on several bio-functions such as autoimmune conditions, angiostasis/angiogenesis and organ-specific tumor metastasis. These properties have rendered chemokines as a promising therapeutic target for several diseases. CXCL10 was primarily recognized in human U937 cells as a histiocytic lymphoma cell line with a monocyte origin as well as in the placenta and spleen as an IFN-γ-inducible chemokine [35,36]. Mob-1 and crg-2 are the rat and mouse homologs of human CXCL10, respectively, that present a 70% and 78% amino acid homology with this CXCL10 [37]. CXCL10 is a low molecular weight protein (10 kDa), similar to other chemokines that are functionally categorized as members of ‘inflammatory’ chemokines [38,39]. Moreover, the ELR tripeptide motif is absent in the CXCL10 structure, further enabling CXCL10 to serve as an inhibitor of angiogenesis or angiostatic chemokines [40]. The CXCL10 gene is located on chromosome 4 and encodes a protein of 98 amino acids [41] in response to external stimuli such as IFN-γ and lipopolysaccharides (LPS). CXCL10 transcription is regulated by a region of 230 nucleotides that contain multiple regulatory components: (a) two regulatory motifs for the nuclear factor kappa-light-chain-enhancer of activated B cells (NF-κB); (b) one site for activator protein 1 (AR); (c) one site for the interferon-stimulated response element (ISRE); and d) one site for the binding of heat shock (HS) factors [42]. The shared receptor of CXCR3 serves as a receptor for three IFN-γ-inducible chemokines, CXCL9, CXCL10 and CXCL11. CXCR3 contains two different isoforms known as CXCR3-A and CXCR3-B and studies have demonstrated that each CXCR3 isoform regulates different cellular functions. The CXCL10/CXCR3-A axis contributes to CXCR3 activation as well as in chemotaxis and the proliferation of various cell types [43,44]. The CXCL10 attachment to CXCR3-B contributes to migration, proliferation inhibition and apoptosis induction [45,46,47]. Furthermore, CXCL10 might serve as an antiangiogenic/antitumor protein [45,48]. The prompt CXCL10 biological activities are not completely known except its active involvement in several physiological and pathological processes such as the specific chemo-attraction of macrophages, monocytes and activated T and NK cells [46,47,48], the modulation of T cell development and function [49,50], the in vitro inhibition of bone marrow progenitor cell formation [51], the facilitation of T cell adhesion to endothelial cells [52], the NK cell migration, NK cell-mediated cytolysis and anti-angiogenesis (including antitumor angiogenesis) [53] as well as further chemotactic and mitotic effects on the vascular smooth muscle cells [51].

## 3. CXCL10 Signal Transduction

It has also been well-evidenced that tumor necrosis factor (TNF)-α and IFN-γ are able to act synergistically for CXCL10 expression by various cell types such as keratinocytes and hepatocytes. These TNF-α and IFN-γ synergistic effects are performed in the NF-κB and ISRE elements located within the CXCL10 promoter [54,55]. In vitro cultures of pancreatic cancer cells revealed that cholecystokinin (CCK8) increased CXCL10 levels through the regulation of the NF-κB activation. Despite this action being blocked by pyrrolidine dithiocarbamate (PDTC), an NF-κB inhibitor, via the inhibition of NF-κB-α degradation, a further induction of CXCL10 expression has been observed [56]. The activation of NF-κB and the CCK8-induced expression of CXCL10 are mediated by protein kinase C (PKC) as well as elevated intracellular calcium levels [56,57]. Several studies have reported that keratinocytes may produce CXCL10 in a concentration-dependent and time-dependent manner in response to TNF-α and IFN-γ, probably mediated by the activation of the protein kinase C (PKC) pathway [58]. Similarly, primary human kidney mesangial cells may also produce CXCL10 via an NF-κB-dependent pathway (through the cooperation between the ISRE and NF-κB sites on the CXCL10 promoter) in response to TNF-α/IFN-γ [59]. Both TNF-α and IFN-γ use signal transducers and an activator of transcription 1α (STAT1α) and NF-κB, respectively, for the induction of CXCL10 by human fibroblastic cell lines [60] and lipopolysaccharide-stimulated (LPS) Kupffer cells [61]. Zohar et al. [31] recently demonstrated that CXCL9 and CXCL10 may promote T helper type 1 (Th1)-T helper 17 (Th17) cell orientation and locomotion along with their polarization throughout the activation of various signal transducers and activators of transcription (STAT) family members including STAT1, STAT4 and STAT5, leading to further inflammation perpetuation. In an inverse fashion, CXCL11 exhibits a higher binding affinity for CXCR3 and fork-head box P3 (FOXP3)-negative interleukin 10 (IL-10)-expressing T regulatory 1 (Tr1) cells and interleukin 4 (IL-4) production by Th2 cells via STAT3 and STAT6 activation, which have been proven to suppress inflammatory responses [31]. The reverse functions of the CXCR3 agonists seem to stem from biased signaling, a feature of the G-protein-coupled receptors (GPCRs) that could further lead to the activation of G-protein-dependent signals, types of signals that are achieved by *β*-arrestin 2 recruitment [31]. These types of biased allosteric agonists that share CXCR3 could selectively activate *β*-arrestin or G-protein-dependent signaling and thus may serve as immunotherapy targets [62].

## 4. CXCL10 and CAD

Coronary artery disease is defined as a chronic inflammatory process accompanied by atherosclerotic plaque formation [63]. A significant correlation was reported between the serum levels of CXCL10 and the severity of coronary artery disease, suggesting a potential role for CXCL10 in both angiogenesis and angiostasis; phenomena that occur during the development of collateral circulation.

### 4.1. The Role of CXCL10-Expressing Cells in CAD

CXCR3-expressing monocytes/macrophages, Th1 cells, NK cells and cytotoxic T cells (CTCs) contribute to atheromatous plaque formation, ulceration, rupture [38] and further thromboses that eventually lead to an acute coronary syndrome. Endothelial dysfunction and the concomitant increased vascular permeability can induce the expression of adhesion molecules by endothelial cells and leukocytes and elevated low density lipoproteins (LDLs) that in turn lead to the development of atherosclerosis [38]. The accumulation of oxidized LDL causes the recruitment of endothelial cells and macrophages toward the intima from the media. The recruited endothelial cells secrete a broad range of pro-inflammatory cytokines and chemokines (including CCL2, fractalkine, CXCL9, CXCL10 and CXCL11) in response to the plasma-oxidized LDL levels, attracting further monocytes that differentiate into dendritic cells or macrophages. Finally, macrophages are converted into foam cells post-accumulation of the oxidized LDL. The population of T lymphocytes that migrate into the intima preserves their capabilities whereas dendritic and NK cells promote the induction of cluster differentiation 4 (CD4) expression on the Th1 phenotype, whichin turn consists of the most abundant T cell type in human atherosclerotic plaques [64]. CXCR3 is a primary requirement for the generation of Th1 cells [65]. Similar to NK cells, Th1 cells can also produce IFN-γ that serves as a factor for Th1 polarization, activating the pro-inflammatory M1 macrophages and further inducing apoptosis.

**Figure 1:**
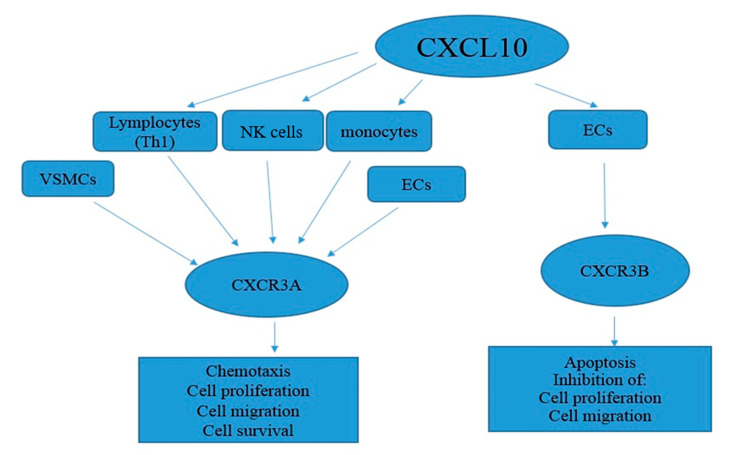
The effect of CXCL10 on CXCR3 isoforms in human tissues. A schematic overview of the CXCR3 isoform expression in human cells post-CXCL10 binding. The isoform of the CXCR3-A receptor has been identified and is known as an angiostatic receptor. CXCR3-A is expressed by means of T lymphocytes (Th1), monocytes, NK cells, VSMCs and endothelial cells (low expression stages). After the binding of CXCL10, the CXCR3-A receptor mediates the cell features including chemotaxis, cellular proliferation, migration and survival. A second isoform, known as CXCR3-B, is recognized in binding CXCL10. This isoform is generally expressed by means of the endothelial cells and is known for its antiangiogenic properties. This encompasses cellular apoptosis and the inhibition of mobile proliferation and migration.

The athermanous plaques formed within the arterial walls consist of accumulated lipids, fibrous connective tissues, macrophages and cellular debris yielded from the cytolytic actions of the oxidized LDL, NK cells, IFN-γ and cytoxic T lymphocytes (CTL) on macrophages, foam cells, vascular smooth muscle cells (VSMCs) and finally endothelial cells. Extracellular matrix (ECM) proteins create a fibrous cap generated by VSMCs leading to further plaque stabilization (Figure 1). Pro-inflammatory M1 macrophages produce matrix metalloproteinases stimulated by IFN-γ that further promote fibrous cap degradation, rendering the plaque vulnerable to rupture.

CXCL10 can be expressed by a wide range of cells such as endothelial cells, smooth muscle cells and macrophages during atherosclerotic plaque formation in both preclinical and clinical conditions [66,67]. The suppression of CXCL10 bioactivity in APOE-deficient mice resulted in a more stable plaque phenotype characterized by a reduced macrophage activation and an increased smooth muscle cell and collagen accumulation [68]. The exact mechanistic part of CXCL10 contributing to the development of atherosclerotic plaques and their subsequent destabilization has not been well-clarified as the plasma concentrations of CXCL10 tend to be elevated in patients with more vulnerable plaque phenotypes [29]. Th1, NK and CTL cell numbers might be increased during unstable plaque formation whereas the number of anti-inflammatory regulatory T (Treg) cells [69] is reduced significantly. More recent studies demonstrated that the relative number of Treg cells was decreased, further inhibiting their actions in CAD patients [70,71]. In CXCL10-knocked out/apolipoprotein E-deficient mice, the atherosclerosis progression was shown to be well-correlated with enhanced Treg cell numbers and their subsequent effect on the reduction of atherosclerotic lesion formations [72].

### 4.2. Mechanism of the CXCL10 Action in CAD

Qiuet al. [73] demonstrated that immune cells along with inflammation might play a fundamental role in the development and progression of vascular atherosclerosis. The effects of CXCL10 expression on cell surface adhesion molecules, inflammatory cytokines and chemokines have been investigated, revealing that IL-27 may, remarkably, stimulate the release of CXCL10. Notably, IL-27 has been demonstrated to significantly contribute to several biological activities such as the TNF-α-mediated expression of intercellular adhesion molecule 1 (ICAM-1) and vascular cell adhesion molecule 1 (VCAM-1), pro-inflammatory cytokine IL-6 and chemokines (C-C motif chemokine ligand 5 (CCL5)/CXCL10 from human cardiovascular endothelial cells). The production of IL-6, CCL5 and CXCL10 by endothelial cells is concealed in specific signaling particle inhibitors, implying that the secretion of such mediators through human coronary artery endothelial cells might be moderated via the c-Jun N-terminal kinase, p38 mitogen-activated protein kinase and NF-κB signaling. These results provide vital information regarding the effect of CXCL10 and IL-27 on the TNF-α-mediated activation of human coronary artery endothelial cells during vascular atherosclerotic lesion formations [73]. In another study, Keeley et al. [74] evaluated the plasma levels of angiogenic and angiostatic chemokines to evaluate if there was an association with both the presence and extent of coronary collaterals in patients with chronic ischemic heart disease (IHD). Elevated levels of IFN-γ, hypertension and diabetes proved to be associated with the absence of collaterals (receiver operating characteristic (ROC) area: 0.91). When analyzed according to the extent of collateralization, higher Rentrop scores were significantly correlated with an increased concentration of CXCL1 as a biomarker of angiogenesis and inversely with decreased concentrations of angiostatic ligands such as CXCL9, CXCL10 and CXCL11 in combination with IFN-gamma. The plasma levels of these chemokines are well-correlated with both the presence and extent of spontaneous coronary artery collateral visualization and may be mechanistically involved in their recruitment [74]. In another study, Oliveira and co-workers [75] evaluated both the messenger ribonucleic acid (mRNA) gene and the protein expression of CCL2, CXCL8, CXCL9, CXCL10, IFN-ɤ and IL-10 in vitro along with CCR2 and CXCR3 expression in peripheral blood mononuclear cells (PBMCs) in CAD patients in comparison with healthy subjects in either the presence or absence of oxidized LDL (oxLDL). Patients suffering from CAD revealed higher contents of CCL2, CXCL8, CXCL9, CXCL10 and IFN-ɤ mRNA. The CAD patients exhibited a higher expression of CCL2 and CXCL8 mRNA levels compared with the control subjects in response to oxLDL. Elevated levels of CCL2 and CXCL8 were also detected in the supernatants of theoxLDL-stimulated pBMCs from the CAD patients when compared to controls.

CAD patients have also displayed a higher percentage of constitutive CCR2+ and CXCR3+ cells after stimulation with oxLDL. A lower IL-10 mRNA production was observed in patients with signs of unstable angina (UA) when compared with those who were exhibiting symptoms of stable angina (SA). Overall, these data suggest that the pBMCs of CAD patients are able to produce higher concentrations of chemokines and cytokines; specifically, chemokines that are involved in the regulation of monocyte/lymphocyte migration and further accumulation in atherosclerotic lesions [75]. It has also been reported that in individuals who underwent coronary artery bypass grafting, the plasma levels of CXCL9, CXCL10 and CXCL11 appeared to be immediately elevated post-heparin administration and significantly diminished post-heparin reversion with protamine. These effects were proven to be independent of either detectable amounts of circulating IFN-ɤ or the IFN-ɤ inducer and interleukin12 (IL-12). These data were in accordance with other previous studies that demonstrated that heparin inhibits the IFN-ɤ-dependent production of the chemokine ligands CXCL9, 10 and 11 in the organ culture of atherosclerotic coronary arteries. Beyond the reduction of chemokine secretion, heparin rapidly displaced membrane-associated CXCL10 from cultured endothelial cells that failed to express CXCR3 and also reduced the CXCL10-dependent trans-endothelial migration of T helper cells under the conditions of regular shear stress. The administration of heparin to immunodeficient mice decreased both the recruitment and infiltration of memory T cells within human coronary arteries. Despite an inhibition of the IFN-ɤ responses, heparin has been proven to display further immune-modulatory effects via competition of the respective attachment to CXCL9, CXCL10 and CXCL11 on endothelial cells. The impaired transport of CXCR3-expressing Th1 cells in atherosclerotic arteries may possibly contribute to the therapeutic efficacy of heparin in inflammatory arterial diseases whereas heparin non-anticoagulant derivatives may represent a novel anti-inflammatory strategy [76]. In addition, apolipoprotein E (APOE)/Cxcl10/mice fed with a Western-style diet for either 6 or 12 weeks demonstrated a significant atherogenesis inhibition compared with APOE (−/−) controls, revealing the role of CXCL10 in atherosclerosis progression [72]. Immunohistochemical studies have reported a reduced accumulation of CD4+ T cells whereas a quantitative reverse transcription polymerase chain reaction (RT-PCR) analysis demonstrated a remarkable reduction in the mRNA of CXCR3 in the atherosclerotic aortic arches. However, regulatory T cell (Treg) numbers and their activity were enhanced, a fact that supports a potential functional role for CXCL10 contribution on the recruitment of T effector cells in atherosclerotic lesions by modulating the local balance of the effecter and the regulatory arms of the immune system [72]. Rothenbacher and colleagues [77] demonstrated higher CXCL10 and CXCL8 and lower CCL5 levels in coronary heart disease patients compared with age- and gender-matched controls.

Positive correlations have also been observed between CXCL8, CXCL10 and multiple acute-phase proteins or inflammation-associated cytokines. A relation between CXC10 plasma levels, viscosity and ICAM-1 has been observed. A study was indicative for the absence of a universal and systemic upregulation of chemokines in CHD-associated inflammation as there was an up regulation of CXCL10 and CXCL8 compared with a down regulation of CCL5. Moreover, a clear disease association for CCL2, CCL4 or CCL11 has not been reported [77]. Zuojun and co-workers demonstrated that CXCL10 plays an important role in intimal hyperplasia as siRNA-mediated CXCL10 silencing inhibited aberrant VSMC hyperplasia and reduced restenosis in atherosclerotic patients [78,79]. In vitro studies of human coronary artery smooth muscle cells (HCASMCs) performed by Chang [80] showed a functional role for plasma-circulating CXCL10 and IL-17 in the development of vascular calcification and revealed its underlying mechanism in cardiovascular disease. HCASMCs treated with CXCL10 and IL-17 developed a phenotype that could promote vascular calcification in a path similar to the bone morphogenetic protein (BMP) 6 autocrine pathway. Furthermore, the BMP6 autocrine stimulation in CXCL10 and IL-17-treated HCASMCs could up regulate the activation of smad1/five-runx2 signaling and increase the bone matrix-related proteins including the osteopontin, osteocalcin and alkaline levels [80]. Lupieri and co-workers [81] demonstrated an unexpected cellular cross-communication in the arterial endothelial cells where a phosphatidylinositide 3-kinase (PD3K)-dependent T cell response resulted in CXCL10 production by smooth muscle cells inhibiting endothelial repairs. Consequently, the inhibition of PI3Kγ and the IFN-γ/CXCL10 axis may serve as novel targets for endothelial repair inductions.

### 4.3. CXCL10 Implications in CAD

The plasma level of CXCL10 has also been reported to be increased in CAD patients [82,83]. Ardigo and colleagues [84] demonstrated that only the CXCL10 serum levels among other chemokines (CCL11, CCL3, CCL2, CCL8, CCL7, CCL13) were significantly increased in CAD patients compared with thecontrol individuals. The background pathophysiologic mechanisms of plaque rupture may involve factors acting locally in the plaque whereas their levels might be independent of the systemic circulation. The elevated CCL4, CXCL16, CXCL8 and CXCL10 circulatory levels within the first week of a percutaneous coronary intervention (PCI) were observed to be positively correlated with the severity of myocardial damage in patients with a first-time ST-elevation myocardial infarction (STEMI) [85]. Recently, a study has evaluated the potential association between the serum levels of CXCL10 and CXCL12 and the degree of coronary artery occlusion in 88 high-risk CAD patients who underwent coronary angiography. A significant correlation between the serum levels of CXCL10 and CXCL12 and the severity of the coronary artery occlusion was observed, further suggesting a potential role of CXCL10 in the processes ofangiogenesis and angiostasis during the development of collateral circulation [86]. In another study, a monoclonal anti-IFNAR1 therapy for four weeks resulted in a reduction of CXCL10 levels, leading to further collateral artery growth stimulation in mice [87]. Niki et al. [82] investigated the potential relationship between coronary atherosclerosis and chemokine family members from either CXC/CC group such as CXCL10, CCL2 and CCL5 (RANTES) or high-sensitivity C-reactive protein (hsCRP), an already established marker of atherosclerotic disease. A group of 28 patients underwent coronary angiography and were further divided into CAD-negative or -positive, respectively. Interestingly, the CAD-positive patients demonstrated higher plasma concentrations of CXCL10 in the aorta as well as significantly higher transcoronary concentration gradients of circulating CXCL10 whereas no significant differences ofCCL2, CCL5 or hsCRP were observed in the aortic plasma concentrations or transcoronary concentration gradients between the CAD-positive and -negative groups. Similarly, another study also showed that both the aortic plasma concentrations and transcoronary concentration gradients of CXCL10 were correlated with the Gensini score in contrast toCCL2, CCL5 and serum hsCRP, suggesting that CXCL10 could serve as a good surrogate marker for the diagnosis of CAD [82,83,84,85,86,87]. In another study, the effects of an antihypertensive treatment with telmisartan on the expression of CXCL10, TNF-α and CCL2 inflammatory markers in CAD patients wereevaluated. The patients in the telmisartan group were compared witha matched control group including 31 hypertensive patients without the telmisartan treatment and they were followed up for about two years. The serum samples for 26 cytokines and chemokines both at the beginning and at the end of the follow-up were collected along with baseline scores of coronary artery calcification (CAC). The CCL2 level was significantly elevated in both the telmisartan and control groups whereas the CXCL10 and TNF-α levels were significantly decreased in the telmisartan patients compared withthe control. These findings suggest that althoughtelmisartan remarkably reduced the blood pressure in patients with atherosclerosis and arterial hypertension within a short duration of time, the inflammatory status of these patients remained largely unchanged, revealing a possible but not clear impact of telmisartan on the regulation of the pro-inflammatory and anti-inflammatory mediators in the pathogenesis of CAD [88]. Kawamura and co-workers [79] evaluated the chemokine receptor membrane expression (CCR2/CCR5/CXCR2/CXCR3) along with the plasma concentrations of CCL2 in 55 CAD patients who underwent percutaneous transluminal coronary angioplasty (PTCA) and 20 patients without a significant coronary stenosis according to the results of a coronary catheterization. The CAD patients were divided into three groups: 20 displayed de novo stenosis, 15 with restenosis and 20 without restenosis. The lymphocytes of the CAD patients without stenosis expressed significantly lower CXCR3 than the patients with stenosis. The elevated plasma level of CXCL10 was associated with a compensatory decreased CXCR3 expression in the lymphocytes but not in the monocytes, proposing a mechanism of monocyte signaling induction via high plasma levels of CXCL10 (Figure 1). Consequently, CXCR3 and CXCL10 chemokineligand and receptor interactions on monocytes may affect the development of coronary restenosis but not the de novo stenosis in patients with CAD [79]. A study performed by Altara et al. [89] demonstrated that CXCL10 was elevated in cardiovascular diseases in parallel with an increased cardiac infiltration of pro-inflammatory Th1 and cytotoxic T cells. CXCL10 is a chemoattractant for these T cells and a polarizing factor for the pro-inflammatory phenotype. Thus, CXCR3 targeting may constitute a promising therapeutic approach for cardiac inflammation management [89].

## 5. CXCL10 Targeting in Various Cardiovascular Diseases

The CXCR3 receptor and its related agonist CXCL10 constitute promising drug targets for the therapy of different cardiovascular disorders. The plasma level of CXCL10 can be elevated in cardiac inflammations along with aninduced Th1 cell polarization/infiltration to the myocardial tissues. The CXCR3 receptor plays a paramount role in recruiting different white blood cells including monocytes, effector lymphocytes and CTL cells in the heart [38,90]. Peroxisome proliferator-activated receptor (PPAR)-γ agonists are considered to bepotentially promising pharmacological agents for the inhibition of CXCL9, CXCL10 and CXCL11 in patients as PPAR-γ agonists have exhibited potent inhibitory effects against their expression in vitro [91]. Pioglitazone could also serve as a favorable cardiovascular protective agent despite its contraindication in heart failure patients due to the exacerbation of fluid retention [96,97,98,99]. Sildenafil was also introduced as another potential therapeutic agent that coulddecrease CXCL10 production inboth protein and mRNA levels in human cardiac myocytes and further attenuate the circulating CXCL10 in diabetic cardiomyopathy patients [92]. There is substantial evidence that sildenafil has protective impacts against adverse cardiac remodeling [93]. CXCL9, CXCL10 and CXCL11 share CXCR3 as a common receptor; however, these agonists may activate opposing responses due to a biased signaling pathway that is a specific feature of the G-protein-coupled receptors [38,94,95]. Although the CXCL9/CXCL10/CXCR3 axis activates effectorTh1, the CXCL11/CXCR3 axis can induce an immunotolerant state defined by T lymphocyte polarization into T regulatory type 1 (Tr1) lymphocytes that are able to produce anti-inflammatory IL-10 cytokines [94,95]. Similar ligands have been designed that exert CXCL11-like actions following an attachment to CXCR3 [62] but their applications on cardiovascular diseases in experimental rodent models has yet to be elucidated, a fact that may be due to the short in vitro half-life of CXCL10. A stabilized form of CXCL11 has been developed by Zohar et al. by producing a fusion protein in which CXCL11 was linked to immunoglobulin G1(IgG1) [31,38,62,63,64,65,66,67,68,69,70,71,72,73,74,75,76,77,78,79,80,81,82,83,84,85,86,87,88,89,90,91,92,93,94,95,96,97,98,99,100]. The specific targeting of CXCR3 may possibly be more effective for the therapy of chronic cardiac inflammation in combination with approaches enhancing Treg numbers and activity, which are generally reduced in cardiovascular diseases. It has been well-established that Treg cells demonstrate immunosuppressive and anti-inflammatory properties in various experimental models of CAD. BXL-01002q, the vitamin D receptor, has also been reported to inhibit IFN-γ and TNF-α-induced CXCL10 secretion by fetal human cardiac myocytes. It also reduces CXCL10 protein secretion and gene expression by CD4+ T cells [101]. The underlying mechanism of the vitamin D receptor agonist involvement in Treg along with the reduction of CXCL10 in patients suffering from heart failure needs to be further delineated. In addition, the activation of CXCL10/CXCR3 receptors may induce the recruitment of T lymphocytes and the perpetuation of mucosal irritation. Subsequently, the reduction of plasma CXCL10 by atorvastatin may also represent another therapeutic approach for Crohn’s disease inthe future [102].

## 6. CXCL10 and Immune Responses in the COVID-19 Vaccination

Immune responses to human immunodeficiency virus (HIV) ALVAC, Ade5 vaccines and to the yellow fever vaccine have previously been described [103,104,105,106] In these vaccines, serum cytokines featuring CXCL10, IFN-g and IL-15 constitute important drivers of inflammation and innate immunity that play a significant role in the creation and maintenance of adaptive immunity in response to both the infection and vaccination. CXCL10 promotes the chemotaxis of CXCR3+ cells, which are mainly activated T and B lymphocytes. A new mechanism by which IL-15 indirectly acts on dendritic cells and macrophages/monocytes to induce the secretion of CXCL10 by IFN-g has recently been proposed [107]. Serum CXCL10 levels, an innate signature linked to IFN and CXCL10-related genes, were found to be associated with higher vaccine-induced antibody titers [108]. The severity of COVID-19 disease is related to an uncontrolled inflammation and the ensuing cytokine storm. Indeed, an increase in multiple type 2 effectors IL-5, IL-8, eosinophils, immunoglobulin E, type 2 antibody isotype IgE and CXCL10 were found in severe form of the disease and continued to increase during the course of disease [109,110]. However, an early but transient inflammatory cytokine response with increased CXCL10, IFN-g, IP-10/, IL-6 and CRP was observed at day 2 and returned to baseline levels by day 8 following a COVID-19 vaccination with BNT162b2 mRNA (Pfizer/BioNtech). The authors of this report wondered whether this early cytokine/chemokine response with increased CXCL10, IL-15 andIFN-g could be used to monitor effective vaccinations and/or as a guide to optimize the efficacy of the mRNA vaccination [111]. Extremely rare cases of acute myocardial infarction and Kounis syndrome have been reported in association with COVID-19 disease and the COVID-19 vaccination including BNT162b2 (Pfizer–BioNTech) [112], the Covishield vaccine [113,114], Sinovac (Coronovac) [115], Moderna [112,113,116] and AstraZeneca [117]. Whether these rare associations are the result of CXCL10 and other chemokines needs to be elucidated.

## 7. Conclusions

According to the most recent scientific evidence, CXCL10 is markedly involved in the pathogenesis of cardiovascular diseases [118]. The degree of involvement is varied from the development and maintenance of hypertension to cardiac remodeling. Notably, targeting of the CXCL10/CXCR3 axis in addition to cardiac inflammation may constitute a new pharmacological approach for either heart failure or CAD therapy supplementary to present drugs that typically target the sympathetic or renin-angiotensin system or platelets [119]. This requires the selective targeting of almost all critical immune pathways via the preservation of the protective and homeostatic immune actions [120]. An antigen-specific modulation of the immune system, for example, through the systemic delivery of nanoparticles covered by disease-relevant peptides bound to a major histocompatibility complex class II (pMHCII) to develop endogenous antigen-specific Tregs may be an alternative therapeutic strategy. A targeted regulation of the immune response may be achieved by the administration of a low dose of IL-2 to promote endogenous Tregs. The development of selective evasins or evasin-like peptides that may be differentially expressed that bind and even neutralize CXCL9/CXCL10 versus CXCL11 could offer an interesting therapeutic alternative to limit the pathogenic and preserve the regulatory CXCR3 functions. The early cytokine/chemokine signature featuring IL-15, IFN-g and CXCL10 may be involved in the pathogenesis of myocardial damage, myocardities and thromboses [110,121] but may also be involved to monitor effective vaccinations and as a guide to optimize the efficacy of the mRNA vaccination [111].

## Data Availability

Not applicable.

## Abbreviations

ACS	Acute coronary syndrome
APOE	Apolipoprotein E
BMP	Bone morphogenetic protein
CVD	Cardiovascular disease
CCK8	Cholecystokinin 8
CD4	Clusterof differentiation 4
CAC	Coronary artery calcification
CAD	Coronary artery disease
CCL5	C-C motif chemokine ligand 5
CHD	Coronary heart disease
CTCs	Cytotoxic T cells
CTL	Cytotoxic T lymphocyte
CXCL10	CXC motif chemokine ligand 10
COVID-19	Coronavirus disease
ELR	Enzyme-linked receptor
ECM	Extracellular matrix
FOXP3	Fork head box P3
GPCRs	G-protein-coupled receptors
HCASMCs	Human coronary artery smooth muscle cells
ICAM-1	Intercellular adhesion molecule 1
IHG	Ischemic heart disease
IFN	Interferon
IL-4	Interleukin 4
IgG1	Immunoglobulin G1
IP-10/CXLC10	Induced protein 10/CXLC10
ISRE	Interferon-stimulated response element
INSRE	Interferon-stimulated response element
LPS	Lipopolysaccharides
LDL	Low density lipoprotein
mRNA	Messenger ribonucleic acid
NK	Natural killer
NF-κB	Nuclear factor-κB
PBMC	Peripheral blood mononuclear cells
pMHCII	Peptides bound to major histocompatibility complex class II
PTCA	Percutaneous transluminal coronary angioplasty
PPAR	Peroxisome proliferator-activated receptor
PDTC	Pyrrolidine dithiocarbamate
PKC	Protein kinase C
PD3K	Phosphatidylinositide 3-kinase
RANTES	Regulated upon activation, normal T cell expressed and secreted
RT-PCR	Reverse transcription polymerase chain reaction
ROC	Receiver operating characteristic
STAT1α	Signal transducer and activator of transcription 1α
Treg cells	Regulatory T cells
TIV	Trivalent influenza vaccine
ΤΡ1	T regulatory type 1
TNF-α	Tumor necrosis factor α
VCAM-1	Vascular cell adhesion protein 1
VSMC	Vascular smooth muscle cells
WBC	White blood count
